# Investigation of long-term survival outcomes and failure patterns of patients with nasopharyngeal carcinoma receiving intensity-modulated radiotherapy: a retrospective analysis

**DOI:** 10.18632/oncotarget.13564

**Published:** 2016-11-24

**Authors:** Wei Zhao, Hao Lei, Xiaodong Zhu, Ling Li, Song Qu, Xia Liang

**Affiliations:** ^1^ Department of Radiation Oncology, Cancer Hospital of Guangxi Medical University, Nanning, 530021, China; ^2^ Department of Radiation Oncology, Hubei Cancer Hospital, Wuhan, 430079, China

**Keywords:** failure pattern, intensity-modulated radiotherapy, long-term outcome, nasopharyngeal carcinoma, retrospective analysis

## Abstract

Intensity-modulated radiotherapy (IMRT) has replaced the conventional radiotherapy (2D-RT) and improved clinical efficacy in Nasopharyngeal Carcinoma (NPC) patients. In the present study, we retrospectively analyzed the clinical characteristics of patients with NPC treated with IMRT to assess the long-term survival outcomes and failure patterns. Of the 527 patients, One hundred and twenty-one patients experienced treatment failure, 86 patients developed distant metastases, and 12 patients developed a second primary tumor. The local and regional recurrence rates were 31.4% and 14.0%, respectively. The 5-year overall survival (OS), progression-free survival (PFS), local recurrence-free survival (LRFS), regional relapse-free survival (RRFS), and distant metastatic relapse-free survival (DMFS) rates were 80.9%, 75.6%, 91.7%, 96.2%, and 83.0%, respectively. The 5-year LRFS rates of Stage T1-4 patients were 100.0%, 93.1%, 92.0%, and 85.8%, respectively. The 5-year DMFS rates of Stage N0-3 patients were 95.0%, 86.1%, 79.5%, and 67.2%, respectively. Multivariate analysis showed age and T-stage were independent predictors of OS, T-stage was an independent predictor of LRFS, and age and N-stage were independent predictors of PFS and DMFS.

In summary, the improved treatment results with IMRT are primarily due to the achievement of a higher local tumor control rate and OS in NPC patients. However, distant metastasis was the most commonly observed failure pattern after treatment. These results provide deep insights about the value of IMRT in the treatment and prognosis of NPC patients.

## INTRODUCTION

NPC is the most common malignant head and neck tumor in Asian patients, particularly in southern China. Considering the high sensitivity of radiotherapy, IMRT has exhibited improved clinical efficacy in NPC patients, and has hence widely replaced the conventional radiotherapy, including two-dimensional radiation therapy technology and three-dimensional conformal radiotherapy technology. From 1996 to 2000, the 5-year OS of 2,687 patients who underwent conventional two-dimensional radiotherapy was 75% [1]. Moreover, the 5-year disease-specific survival rate (2005–2010) of 444 patients treated with IMRT was approximately 85% [2]. Thus, IMRT has become the standard technology for the treatment of NPC for improving local control and protecting the surrounding normal tissue. However, to our knowledge, studies with a large sample, long-term follow-up, and various disease stages among patients with NPC are rare. In the present study, we retrospectively analyzed the clinical characteristics of patients with biopsy-proven, non-metastatic NPC treated with IMRT from January 2007 to December 2011 in our hospital to assess the long-term survival outcomes and failure patterns.

## RESULTS

### Follow-up results

At the end of the study period, the follow-up rate was 98.3% and median follow-up time was 38 months (range, 4–97 months). Moreover, 18.4% (97/527) patients died, 23.0% (121/527) exhibited treatment failure, and 2.3% (12/527) developed second primary tumors (SPTs) SPTs. Among the patients with treatment failure, 71.1% (86/121) developed distant metastasis, 31.4% (38/121) developed local recurrence, and 14.0% (17/121) developed regional lymph node recurrence, 57.0% (69/121) developed simple distant metastasis (median failure duration, 14 months), 21.5% (26/121) developed simple local recurrence (median failure duration, 23.5 months), 7.4% (9/121) developed both local recurrence and metastasis (median failure duration, 19 months), 5.8% (7/121) developed regional lymph node recurrence (median failure duration, 34 months), 5.8% (7/121) developed both local recurrence and metastasis (median failure duration, 9 months), 1.7% (2/121) developed both regional lymph node recurrence and local recurrence (median failure duration, 14 months), and 0.8% (1/121) developed regional lymph node recurrence, local recurrence, and metastasis (median failure duration, 22 months).

### Survival analysis

**Figure 1:**
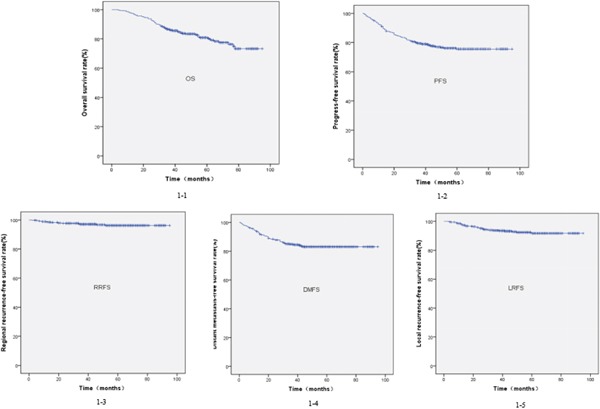
Kaplan-Meier survival curves for the overall survival (1-1), progression-free survival (1-2), regional recurrence-free survival (1-4), distant metastasis-free survival (1-5), and local recurrence-free survival (1-3) rate of all the patients with nasopharyngeal carcinoma

At 1 year, 3 years, and 5 years, the overall OS rates were 97.5%, 86.5%, and 80.9%; the PFS rates were 91.1%, 79.4%, and 75.6%; the LFRS rates were 97.9%, 93.7%, and 91.7%; the RRFS rates were 98.7%, 97.0%, and 96.2%; and the DMFS rates were 93.7%, 84.9%, and 83.0%, respectively. These are shown in Figure 1.

**Figure 2:**
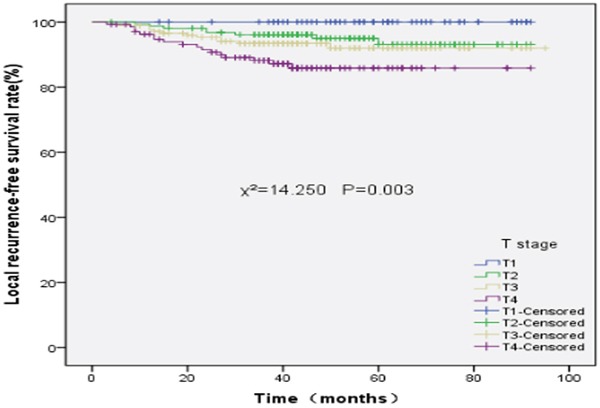
Local recurrence-free survival rates of 527 patients with nasopharyngeal carcinoma at different T stages

The 5-year LFRS rates of stage T1, T2, T3, and T4 disease were 100%, 93.1%, 92.0%, and 85.8%, respectively (χ^2^=14.250, P=0.003). In addition, the 5-year LFRS rates between stage T1 and T2 (χ^2^=3.540, P=0.060), stage T2 and T3 (χ^2^=0.684, P=0.408), and stage T3 and T4(χ^2^=3.264, P=0.071) were not significantly different. However, the 5-year LRFS rates between stage T1 and T3 (χ^2^=4.786, P=0.029), stage T1 and T4(v^2^=9.026, P=0.003), and stage T2 and T4 (χ^2^=6.729, P=0.009) were significantly different. The LRFS survival curves of different T stages are shown in Figure 2.

**Figure 3:**
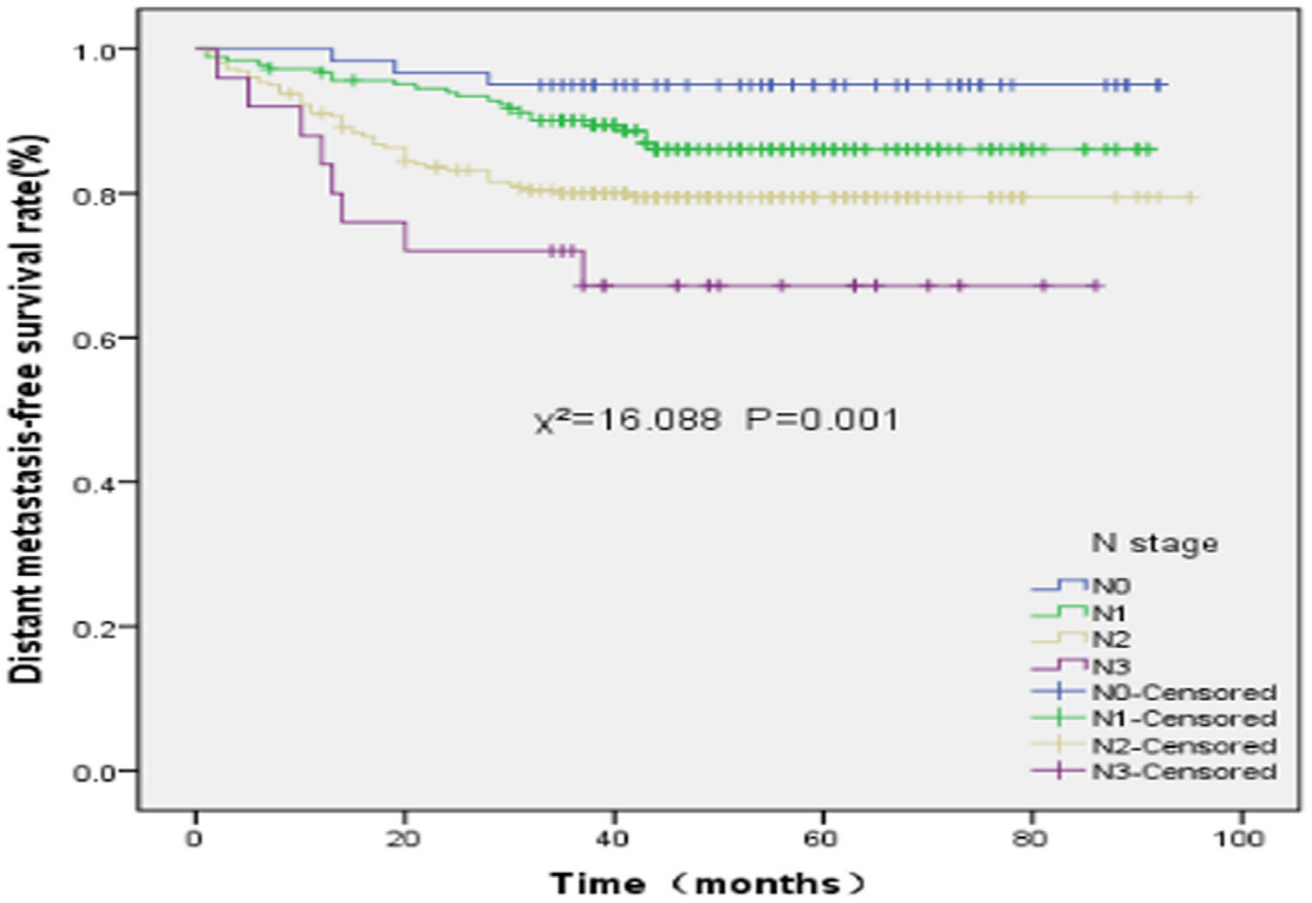
Distant metastasis-free survival rates of 527 patients with nasopharyngeal carcinoma at different N stages

The 5-year DMFS rates of stage N0, N1, N2, and N3 disease were 95.0%, 86.1%, 79.5%, and 67.2%, respectively (χ^2^=16.088, P=0.001). The 5-year DMFS rates between stage N0 and N1 (χ^2^=2.878, P=0.090), stage N2 and N3 disease (χ^2^=2.059, P=0.151) were not significantly different. However, The 5-year DMFS rates between stage N0 and N2 (χ^2^=7.703, P=0.006), stage N0 and N3 (χ^2^=12.659, P=0.000), stage N1 and N2 (χ^2^=4.908, P=0.027), and stage N1 and N3 (χ^2^=8.022, P=0.005) were significantly different. The DMFS survival curves of different N stages are shown in Figure 3.

### Analysis of prognostic factors

Univariate analysis revealed that the application of concurrent, neoadjuvant, and adjuvant chemotherapy did not significantly improve the prognosis of patients. In particular, the T stage and clinical stage were factors that independently influenced the LRFS rate. Moreover, age, T stage, N stage, and clinical stage were the factors that independently influenced the DMFS, OS, and PFS rates (Table 1). Multiple factor analysis indicated that age and T stage independently influenced OS; T stage independently influenced LRFS; and age and N stage independently influenced DMFS and PFS (Table 2).

**Table 1:** Univariate analysis of various clinical endpoints

Characteristic	N	5-year LRFS	*χ*^2^	*P*	5-year RRFS	*χ*^2^	*P*	5-year DMFS	*χ*^2^	*P*	5-year OS	*χ*^2^	*P*	5-year PFS	*χ*^2^	*P*
**Sex**			0.438	0.508		0.648	0.421		1.904	0.168		1.733	0.188		2.809	0.094
Male	394	91.2			95.9			81.9			78.9			73.9		
Female	133	93.2			97.1			86.4			86.4			80.3		
**Age (years)**			2.505	0.114		0.890	0.452		4.317	0.038		10.275	0.001		4.844	0.028
≤44	255	93.9			95.2			86.4			85.6			79.6		
>44	272	89.1			97.5			79.3			75.7			71.3		
**T stage**			14.250	0.003		0.984	0.805		11.975	0.007		22.287	0.000		20.965	0.000
T1	62	100.0			97.6			93.5			93.3			91.2		
T2	156	93.1			96.2			85.8			86.8			77.5		
T3	174	92.0			96.5			83.2			80.2			77.4		
T4	135	85.8			96.0			74.7			65.8			64.5		
**N stage**			5.375	0.146		2.686	0.443		16.088	0.001		14.285	0.003		22.575	0.000
N0	60	94.7			100.0			95.0			90.6			89.8		
N1	184	94.4			96.0			86.1			81.4			80.7		
N2	258	91.1			95.3			79.5			79.3			71.7		
N3	25	71.4			95.7			67.2			67.8			44.8		
**Clinical stage**			12.482	0.006		1.230	0.746		20.046	0.000		27.607	0.000		28.673	0.000
I	18	100.0			100.0			100.0			100.0			100.0		
II	111	95.5			95.8			91.0			87.4			84.3		
III	245	94.0			96.5			84.4			84.3			79.0		
Iva-b	153	82.1			95.7			73.0			67.1			59.8		
**Concurrent chemotherapy**			1.535	0.215		0.922	0.337		0.728	0.394		0.226	0.635		0.570	0.450
No	77	95.9			94.6			86.4			80.9			79.9		
Yes	450	90.7			96.4			82.5			80.8			74.6		

OS, overall survival;PFS, progression-free survival; LRFS, local recurrence-free survival;RRFS, regional recurrence-free survival, DMFS, distant metastasis-free survival

**Table 2:** Multivariate analysis of various clinical endpoints

Variable	HR	95%CI		*P*
		Lower	Upper	
**LRFS**				
T stage	3.676	1.093	12.366	0.035
**DMFS**				
Age	1.584	1.025	2.446	0.038
N stage	1.803	1.025	3.173	0.041
**OS**				
Age	1.887	1.243	2.865	0.003
T stage	2.131	1.153	3.940	0.016
**PFS**				
Age	1.500	1.039	2.165	0.030
N stage	2.009	1.230	3.282	0.005

OS, overall survival; PFS, progression-free survival; LRFS, local recurrence-free survival; RRFS, regional recurrence-free survival, DMFS, distant metastasis-free survival; CI, confidence interval

A total of 38 cases exhibited local recurrence, including 37 with complete nasopharyngeal and neck MRI data of both primary and recurrent NPC. The most common location of the primary NPC was the nasopharyngeal space, parapharyngeal space, and skull base, in that order. Moreover, the most common sites of recurrence of NPC after treatment with IMRT were the nasopharyngeal space, skull base, and parapharyngeal space, in that order. The differences in the location of invasion with primary and recurrent NPC are shown in Table 3.

**Table 3:** Comparison of tumor invasion in adjacent regions between pNPC and rNPC

Tumor invasion site	Invasion in pNPC and rNPC (number)	Invasion in pNPC alone (number)	Invasion in rNPC alone (number)	No invasion in pNPC or rNPC (number)	*P*
Nasopharynx	29	8	0	0	0.008
Oropharynx	1	2	0	34	0.500
Nasal cavity	4	5	3	25	0.727
Parapharyngeal space	10	22	0	5	<0.001
Skull base	19	9	2	7	0.065
Paranasal sinus	5	5	6	21	1.000
Intracranial cavity	7	4	2	24	0.687
Infratemporal fossa	1	2	0	34	0.500
Laryngopharynx	0	0	1	36	1.000
Orbital apex	0	2	1	34	1.000
Masticatory muscles	1	7	2	27	0.180

Abbreviation: pNPC=primary nasopharyngeal carcinoma, rNPC= recurrent nasopharyngeal carcinoma

### Analysis of regional lymph node recurrence

A total of 17 cases exhibited regional lymph node recurrence; all these patients had complete nasopharyngeal and neck MRI data of the primary and recurrent NPC. These MRI data indicated that the rate of invasion of level I lymph nodes in both primary and recurrent cases was 23.5% (4/17), whereas the rate of invasion of level II lymph nodes in primary and recurrent cases were 100% (17/17) and 64.7% (11/17), respectively. In primary and recurrent cases, the rates of invasion of level III lymph nodes were 58.8% (10/17) and 23.5% (4/17); those of level IV lymph nodes were 29.4% (5/17) and 0% (0/17); those of level V lymph nodes were both 11.8% (2/17); and those of parotid lymph nodes were 0% (0/17) and 11.8% (2/17), respectively. In addition, the most common invasion sites of primary tumors included level II and level III lymph nodes, whereas the most common invasion sites of recurrent cases included level II, III, and I lymph nodes. The distribution of the sites of invasion among the lymph nodes in cases of primary and recurrent NPC are shown in Table 4.

**Table 4:** Comparison of tumor invasion in lymph nodes between pNPC and rNPC

Node invasion site	Invasion in pNPC and rNPC (number)	Invasion in pNPC alone (number)	Invasion in rNPC alone (number)	No invasion in pNPC or rNPC (number)	*P*
I	1	3	3	10	1.000
II	11	6	0	0	0.031
III	4	6	0	1	0.031
IV	0	5	0	12	0.063
V	1	1	1	14	1.000

pNPC, primary nasopharyngeal carcinoma; rNPC, recurrent nasopharyngeal carcinoma

### Characteristics of distant metastases

A total of 86 cases exhibited distant metastasis, including 43.0% (37/86) with bone metastasis, 41.9% (36/86) with lung metastasis, 31.4% (27/86) with liver metastasis, 17.4% (15/86) with metastasis in the mediastinal lymph nodes, 11.6% (10/86) with metastasis in the abdominal lymph nodes, 2.3% (2/86) with metastasis in the axillary lymph node, 5.8% (5/86) with metastasis in the brain, and 1.2% (1/86) with metastasis in the pancreas, pericardium, and kidney. Among the cases with metastasis, most of the patients with mediastinal lymph node metastasis also showed lung metastasis, and all the patients with abdominal lymph node metastasis also showed liver metastasis. The characteristics of distant metastasis are shown in Table 5.

**Table 5:** Characteristics of distant metastasis

Site	Total	Disease incidence	Single organ	Multiple organs
Bone	43.0% (37/86)	7.0% (37/527)	19	18
Lung	41.9% (36/86)	6.8% (36/527)	17	19
Liver	31.4% (27/86)	5.1% (27/527)	13	14
Mediastinal lymph nodes	17.4% (15/86)	2.8% (15/527)	2	13
Abdominal lymph nodes	11.6% (10/86)	1.9% (10/527)	0	10
Axillary lymph node	2.3% (2/86)	0.4% (2/527)	1	1
Brain	5.8% (5/86)	0.9% (5/527)	3	2
Pancreas	1.2% (1/86)	0.2% (1/527)	0	1
Kidney	1.2% (1/86)	0.2% (1/527)	0	1
Pericardium	1.2% (1/86)	0.2% (1/527)	0	1

## DISCUSSION

Since it was first clinically introduced in 1990, IMRT has become the first-choice treatment option for the treatment of NPC. In a recent study, Sun et al retrospectively analyzed 868 patients with NPC treated with IMRT, wherein approximately 69.4% patients had phase III-IVa disease, and found that the 5-year Disease Specific Survival (DSS), LRFS, RRFS, DMFS, and PFS rates were 84.7%, 91.8%, 96.4%, 91.8%, and 77.0%, respectively [3]. In the present study, the proportion of patients with phase III-IVa-b disease was approximately 75.5%, and the 5-year OS, LRFS, RRFS, DMFS, and PFS rates were 80.9%, 91.7%, 96.2%, 91.7%, and 75.6%, respectively. Compared with the results of a study on non-IMRT treatment options in our hospital [4], which are similar to those of studies in other research centers [2, 5, 6], the present study indicated that IMRT significantly improved the patient's OS, LRFS, and PFS. Moreover, our study indicated that distant metastasis was the most common cause of treatment failure in patients with NPC, followed by local recurrence and regional lymph node recurrence. Although previous studies on IMRT have reported similar results [33, 7, 8], studies on conventional radiotherapy of NPC have indicated that local recurrence is the main reason for treatment failure [1, 5, 6]. Moreover, we observed that 12 patients with NPC developed SPTs after treatment. In addition, in studies on conventional radiotherapy, the incidence of SPTs was approximately 2.0–5.2% [9–12]. Hence, the incidence of SPTs induced by IMRT and conventional radiotherapy is similar. However, as the follow-up duration of the preset study was relatively short and the incidence of SPTs would increase with an increase in the follow-up duration, physicians should carefully consider the development of SPTs after treatment with IMRT.

Due to the presence of a dose-effect relationship between local control and exposure dose in NPC, the enhancement of the irradiation dose to the tumor could improve the local control rates of patients. Compared to the results of conventional two-dimensional radiotherapy [1, 5, 6], the present study indicated that IMRT significantly improved the local control rate of patients with NPC; in fact, the local control rate would also benefit from the enhancement of the irradiation dose in the tumor and high-risk regions. Furthermore, our study showed that there was no significant difference in the LRFS rate between patients with stage T1 and T2 disease, stage T2 and T3 disease, and stage T3 and T4 disease. In a study of 985 NPC patients who underwent IMRT, Lee et al showed that there was no significant difference in the LRFS rate between patients with stage T1 and T2 disease [13]. Moreover, Chen et al did not show any significant difference in the LRFS rate between stage T2 and T3 patients treated with IMRT [14]. Similarly, a study of 1241 patients treated with IMRT revealed that there was no significant difference in the LRFS rate between stage T1 and T2, and stage T2 and T3 patients [15]. Despite the differences in the sample size and follow-up duration in the above-mentioned studies, the improvement of the treatment effect as a result of advancements in irradiation technology should not be neglected, particularly in terms of dose enhancement in IMRT as compared to dose-restricted areas (e.g., retropharyngeal space, skull base, etc.) in conventional two-dimensional radiotherapy [16]. Therefore, we believe that the reason for the lack of a significant difference in the LRFS rates between adjacent T stages is a result of the improvement of dose application with IMRT as compared to dose-restricted areas in conventional 2D-RT, which is not completely reflected in the 2010 Union for International Cancer Control (UICC) staging system.

The present study indicated that the nasopharynx (78.4%) and skull base (57.8%) are the most common sites of recurrence, followed by the parapharyngeal space, intracranial region, paranasal sinus, nasal cavity, chewing muscles, region below the temporal fossa, orbit, and oropharynx, with rates <10%. This result is similar to that of the study of Li [17] and Ng [18] who showed that the recurrence rates in the nasopharynx and skull base of NPC patients after IMRT were approximately 78.1% and 59.4%, respectively. There are several potential reasons why the recurrent tumors in the nasopharyngeal space, including nasopharyngeal carcinoma stem cells, are not sensitive to radiation and chemotherapy, such as inadequate exposure dose, repopulation of resting stage cells, inaccuracy of target delineation, and unsuitability of radiation treatment plan design; however, further research is needed to obtain a final confirmation. However, for the patients treated with conventional radiotherapy, the most sites of recurrenc is skull base anis the most common site of recurrence in NPC patients treated with conventional radiotherapy [18]; this is reportedly associated with the low dose applied to this region, as a result of the dose limitation to the important organs around the skull base and the dose attenuation in bone [19]. The present study indicated that the skull base the second most common site of recurrence in NPC patients treated with IMRT.

Moreover, our study showed that the most common sites of recurrence in the head and neck lymph nodes include the level II lymph nodes, which is similar to conventional radiotherapy for NPC [20]. This finding was also similar to that observed in the primary lymph node metastasis of NPC—i.e., the level II lymph nodes are the most common sites of invasion in primary NPC [21, 22]. In the present study, there were 2 cases with parotid gland regional lymph node recurrence, which is rare with conventional radiotherapy technology; however, the incidence of parotid gland regional lymph node recurrence has been increasing with the widespread application of IMRT [23, 24]. One reason for this finding is believed to be the under-dosing of subclinical lesions in the parotid gland, in an attempt at overprotection of the parotid gland, which is adjacent to the parapharyngeal space. Moreover, 4 cases exhibited recurrence in the level I lymph nodes. In general, the invasion rate of level I lymph nodes in primary NPC is low, and level I lymph nodes are considered to represent the routine lymphatic drainage path of NPC [21]. Hence, the recurrence in level I lymph nodes is probably associated with the neck lymphatic block and upstream block caused by pipeline fibrosis after radiotherapy [25].

In the present study, distant metastasis was the main cause of treatment failure in patients with NPC after IMRT; bone, lung, and liver were the most common sites for distant metastases. This result is inevitable following the improvement of local control via IMRT, although the enhancement of the local dose for potentially hidden metastatic lesions would be meaningless during the first visit. The study of Lai et al showed that the changes in radiation technology did not significantly affect the control of distant metastasis in patients with NPC, as observed in patients with NPC treated with IMRT or 2D-RT [26]. With regard to the simultaneous development of primary tumors and metastases, current studies indicate that some tumor cells spread from early tumors or precancerous lesions and form hidden tiny lesions, which subsequently proliferate in suitable local conditions. Hence, the improvement of the detection rate of hidden metastasis via modern imaging techniques and laboratory examination methods would influence the treatment choice of patients with NPC and improve the control of distant metastasis and the prognosis. In our study, we also observed that the 5-year DMFS rates of stage N0, N1, N2, and N3 patients were 95.0%, 86.1%, 79.5%, and 67.2% respectively. Thus, with an increase in the N stage, the DMFS rates significantly decreased, particularly in patients with stage N3 who have poor control of distant metastases. Most studies on NPC patients treated with IMRT have exhibited similar results, and stated that the presence of distant metastasis usually indicates poor prognosis. However, in the future, the manner in which the DMFS rates of NPC patients with late N stages can be improved will be essential in improving the therapeutic effect.

Chemotherapy is an effective method for the control of distant metastasis, and may also be involved in radiotherapy sensitization., Concurrent chemotherapy (CCT) has been routinely used in the treatment of locally advanced NPC. In a meta-analysis of clinical research studies on NPC, Zhang et al [27] showed that CCT could improve OS, locoregional control rates, and distant metastasis control rates in locally advanced NPC. However, the conclusion that patients with NPC can benefit from CCT is always based on the results of conventional two-dimensional radiation. At present, the benefits of CCT are unclear in patients with NPC treated with IMRT [28]. Neoadjuvant chemotherapy is considered to be effective for controlling hidden metastatic lesions, although the resultant survival benefit in patients with locally advanced NPC remains controversial. The meta-analysis of Chua et al indicated that, compared with radiotherapy alone, neoadjuvant chemotherapy combined with radiotherapy improved the local control of patients with locally advanced NPC [29]. Moreover, another meta-analysis involving 6 clinical research studies showed that neoadjuvant chemotherapy had no effect on the local control rates of patients with locally advanced NPC [30]. Another meta-analysis on 11 clinical research studies indicated that, compared with concurrent chemoradiotherapy with or without adjuvant chemotherapy, neoadjuvant chemotherapy combined with concurrent chemoradiotherapy failed to yield any survival benefit in patients with NPC [31]. Moreover, we observed that CCT, neoadjuvant chemotherapy, or adjuvant chemotherapy failed to yield any benefit in the OS rate, locoregional control rates, distant metastasis control rate, or PFS rate in NPC patients.

Cox proportional hazards regression indicated that T stage is a significant risk factor for LRFS and OS, N stage is a significant risk factor for DMFS and PFS, and age is a significant risk factor for DMFS, OS, and PFS. Based on a study by Sun et al, T and N stages are significant risk factors for DMFS, PFS, and DSS in patients with NPC, and that a minimal Gross tumor volume (GTV) dose was a significant risk factor for LRFS [3]. The study of Ng also indicated that a minimal GTV dose was a significant risk factor for LRFS [32]. As the purpose of the present study was to primarily evaluate the long-term curative effect and summarize the characteristics of survival, no additional dosimetry analysis was required.

In summary, our study finally describes the improved treatment results with IMRT over two dimensional-radiotherapy (2D-RT) are primarily due to the achievement of a higher local tumor control rate and OS rate in NPC patients. Moreover, Although more work is needed to fully elucidate the long-term side-effect, we have determined that distant metastasis is the most commonly observed failure pattern after treatment. These results provide deep insights about the value of IMRT in the treatment and prognosis of NPC patients.

## MATERIALS AND METHODS

### Patients

We enrolled 527 patients with NPC who were treated at the Cancer Hospital of Guangxi Medical University from January 2007 to December 2011, and met the following criteria: histologically confirmed NPC on nasopharyngeal biopsy; no evidence of metastasis; no previous malignancy or other concomitant malignant disease; no previous treatment for NPC; Karnofsky performance status of ≥70; and completion of radical radiotherapy, without any metastasis during the treatment.

The initial work-up included a complete physical examination, computed tomography (CT) or magnetic resonance imaging (MRI) of the head and neck, histological confirmation of nasopharyngeal lesions, chest radiography or CT, abdominal ultrasonography or CT and single photon emission computed tomography (SPECT) of bone, all of which were used for the exclusion of distant metastases.

The patients included 394 men and 133 women (ratio, 2.9:1), with a median age of 44 years (range, 16–79 years). Patients were staged according to the 2010 Union for International Cancer Control (UICC) staging system. Pathology classification was based on the World Health Organization (WHO) guidelines, with Grade I representing squamous cell carcinoma, Grade II representing non-keratinizing carcinoma, and Grade III representing undifferentiated carcinoma. The baseline data and characteristics of the patients are listed in Table 6.

**Table 6:** Characteristics and treatment factors for the entire series of 527 patients

Characteristics	No. of patients	%
**Sex**		
Male	394	74.8
Female	133	25.2
**Age**		
>44 years	255	48.4
≤ 44 years	272	51.6
**Histological type**		
Non-keratinizing carcinoma	523	99.2
Keratinizing squamous carcinoma	4	0.8
**Tumor stage**		
T_1_	62	11.8
T_2_	156	29.6
T_3_	174	33.0
T_4_	135	25.6
**Node stage**		
N_0_	60	11.4
N_1_	184	34.9
N_2_	258	49.0
N_3_	25	4.7
**Clinical stage**		
I	18	3.4
II	111	21.1
III	245	46.5
IV_a-b_	153	29.0
**Chemotherapy**		
Yes	468	88.8
No	59	11.2
**Concurrent chemotherapy**		
Yes	450	85.4
No	77	14.6
**Neoadjuvant chemotherapy**		
Yes	94	17.8
No	433	82.2
**Adjuvant chemotherapy**		
Yes	310	58.8
No	217	41.2

### Radiotherapy and chemotherapy

All patients were immobilized in the supine position with an individually manufactured precision mask, from the head to the shoulders. Contrast-enhanced CT images were obtained using a CT simulator. After delineation of the target was completed, data were imported into the treatment planning system for treatment design. The prescribed radiation dose to the planning target volume (PTV) including the primary nasopharyngeal gross tumor volume (GTVnx) and involved cervical lymph nodes (GTVnd) was 69.96–74.09 Gy, to the PTV including the high-risk regions (CTV1) was 60–65.1 Gy, and to the PTV including the low-risk regions and neck nodal regions (CTV2) was 51.62–57.6 Gy. IMRT was delivered via 9 fixed-gantry angles with step-and-shoot treatment techniques. All of the patients were treated with 1 fraction daily, for 5 days per week.

During the study period, 468 of the 527 patients received chemotherapy. Of patients with stage II-IVa-b, 41 were treated with radiotherapy alone for economical or personal reasons. Concurrent chemotherapy (CCT) was administered to 112 patients, neoadjuvant chemotherapy (NACT)+CCT was administered to 40 patients, CCT+adjuvant chemotherapy (AC) was administered to 258 patients, NACT+CCT+AC was administered to 40 patients, and NACT or AC was administered to 18 patients.

Patients received a total of 1–8 cycles of chemotherapy. The chemotherapy strategy included CCT with cisplatin (100 mg/m^2^, on day 1, every 21 days) as well as neoadjuvant chemotherapy and adjuvant chemotherapy with PF (cisplatin [80 mg/m^2^] on day 1, 5-FU [750 mg/m^2^] on days 1–4, civ96h, every 21 days), TP (docetaxel [60–75 mg/m] on day 1, cisplatin [60–80 mg/m^2^] on day 1, every 21 days), and TPF (cisplatin [60 mg/m^2^] on day 1, docetaxel [60 mg/m^2^] on day 1, 5-FU [600 mg/m^2^] on days 1–5, civ120h, every 21 days).

### Follow-up and evaluation methods

After the initial treatment, patients were followed up every 3 months during the first 3 years, every 6 months during the next 2 years, and then annually. Chest radiography, abdominal ultrasound, MRI of the nasopharynx and cervical region, and laboratory analysis were performed at each assessment. CT of the chest and abdomen, and SPECT of the entire body were performed every 6 months.

Local recurrence, regional lymph node recurrence, and distant metastasis were confirmed based on pathological examination or imaging findings along with clinical follow-up. SPTs were diagnosed based on the criteria of Warren and Gates [33].

### Statistical analysis

All statistical analyses were performed using the SPSS 22.0 statistical package (SPSS, Chicago, IL). Survival time was calculated from the date of treatment initiation to the date of death or the last follow-up. The χ^2^ test was performed to assess the associations between metastasis and clinicopathological parameters. The Kaplan-Meier method with a log-rank test was used to calculate the survival rate. To assess the effects of clinicopathological parameters on survival, The Cox proportional hazards regression model was used for univariate and multivariate analyses. Factors were included in univariate analysis as follow: sex, age, clinical stage (T stage, N stage), overall IMRT time, chemotherapy (concurrent chemotherapy, neoadjuvantchemotherapy, adjuvantchemotherapy). All statistical tests were two-tailed, and a P value of <0.05 was considered statistically significant.

## Abbreviations

NPC: Nasopharyngeal carcinoma
IMRT: Intensity-modulated radiotherapy
SPT: Secondary primary tumor
OS: overall survival
LRFS: local recurrence-free survival
RRFS: regional relapse-free survival
DMFS: distant-metastatic relaps free survival
PFS: progression-free survival
2D-RT: two dimensional-radiotherapy
GTV: Gross target volume
PTV: Planned target volume
CCT: Concurrent chemotherapy
NACT: Neoadjuvant chemotherapy
AC: Adjuvant chemotherapy
CT: Computed tomography
MRI: Magnetic resonance imaging.
